# The relationship model among parent–child relationship, coping responses and behavioral problems in children with attention deficit hyperactivity disorder

**DOI:** 10.1186/s12888-022-04224-3

**Published:** 2022-09-08

**Authors:** Soulmaz Taghizade, Zohreh Mahmoodi, Atefeh Zandifar, Mostafa Qorbani, Farima Mohamadi, Niloufar Mehrafzoun

**Affiliations:** 1 Student Research Committee, Alborz University of Medical Sciences, Karaj, Iran; 2 Social Determinants of Health Research Center, Alborz University of Medical Sciences, Karaj, Iran; 3 Non-Communicable Diseases Research Center, Alborz University of Medical Sciences, Karaj, Iran; 4 Endocrinology and Metabolism Research Center, Endocrinology and Metabolism Clinical Sciences Institute, Tehran University of Medical Sciences, Tehran, Iran; 5 Social Determinants of Health Research Center, Shahid Beheshti University of Medical Sciences, Tehran, Iran; 6 Kermanshah University of Medical Sciences, Kermanshah, Iran

**Keywords:** Parent–child relationship, Coping responses, Behavioral problems in children, ADHD

## Abstract

**Background:**

Attention Deficit Hyperactivity Disorder (ADHD) constitutes a prevalent behavioral problem. The present study examined the parent–child relationship model and investigated strategies to cope with behavioral problems in children with ADHD.

**Methods:**

This descriptive study selected 300 children with ADHD using convenience sampling. The data collected using the child behavior checklist, the parent–child relationship scale (PCRS), the Billings and Moos Coping Checklist, the socioeconomic status questionnaire, the general health questionnaire-28 (GHQ-28) and a demographic checklist were analyzed in SPSS-25 and LISREL 8.8.

**Results:**

According to the results of the path analysis on the relationship model among parent–child relationship domains, coping responses and children's behavioral problems, parent–child dependency domain (B = 0.22) in the direct path, disease duration (B = 0.085) in the indirect path, and conflicts in the domain of parent–child relationship (B = 0.366) in both direct and indirect paths had the most positive causal effect on behavioral problems. Furthermore, intimacy in the said domain (B = -0.42) had the most negative causal effect in both direct and indirect paths. The extent to which parents used coping responses via the direct path had a positive causal effect on behavioral problems (B = 0.12).

Based on the path analysis test findings in the relationship model among positive parent–child relationship, coping responses and children's behavioral problems, the positive parent–child relationship score had the most negative causal effect via the direct path (B = -0.56). Conversely, the child's age had the highest positive causal effect via the indirect path (B = 0.1) on behavioral problems in children.

**Conclusion:**

Based on findings, there is a causal and significant relationship between the parent–child relationship and the extent to which coping responses are used. It is recommended that training programs be developed to strengthen communication skills, coping responses and problem-solving techniques in parents.

## Background

Childhood plays a key role in humans' comprehensive development and formation of their identity and personality; the circumstances into which children are born determine their exposure to environments that promote or compromise healthy development. Several direct adverse experiences can compromise children's health, development, and well-being during the prenatal and postnatal periods, including persistent poverty, repeated abuse and neglect, negative parental behavior, and family violence [1]. In addition, the majority of conflicts and behavioral disorders in adolescents and adults result from neglecting emotional-behavioural dimensions and failing to properly guide their growth and development in their childhood [2].

As a major neurodevelopmental disorder in children [3], ADHD is characterized by patterns of inattention and hyperactivity-impulsiveness that impair performance or growth. In addition, the symptoms emerging before the age of seven and lasting for at least six months are associated with dysfunction at home and school and relationship problems with playmates [4].

The global prevalence of ADHD among children and adolescents is estimated at 3–7% [5]. About 63 million children and adolescents worldwide suffer from this disorder [6]. The prevalence of this disorder in Iran is insignificantly different from the global average. The differences observed can be explained by differences in the prevalence in different provinces of the country [7].

Behavioral problems in these children include irritability, aggression, opposing behaviors, emotional dysregulation, disappointment, isolation and depression [8]. All these symptoms adversely affect the family life of the patient. Stress levels are higher, and self-esteem is lower in the parents of these children than in the parents of normal children. As a result, these parents experience an intolerable life, and their disappointment, stress, depression, and numerous socioeconomic and physical problems affect their quality of life [9].

Early interventions are essential for children with ADHD. Treatment instructions, environmental recommendations and psychosocial and psychiatric interventions are recommended based on the severity of the disorder in these children [10]. The most common medication for ADHD is stimulants such as Ritalin and Adderall. Of course, non-stimulant drugs such as Strattera or a certain class of antidepressants can also be used for those who do not respond to stimulant medications or experience side effects [6].

The parents and how they deal with their children play a key role in the treatment and quality of life of their affected children. Therefore, it has been recommended that behavioral problems be treated in children in collaboration with their parents and based on family-oriented programs [11]. According to Carnes-Holt, the parent–child interaction is the first point of contact for the child in communicating with the world and an important and vital relationship for creating security and love, consisting of a combination of behaviors, feelings, and expectations which are unique to a particular parent and a particular child [12].

**Fig. 1:**
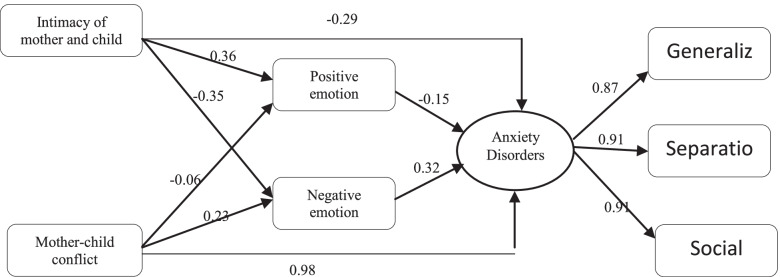
Mother–child relationship and relationships of positive and negative emotions with anxiety disorders [13]

The components of parent–child interactions constitute the predictors of externalizing problems in children. For example, according to the conceptual model of mother–child relationships and relationships of positive and negative emotions with anxiety disorders (Fig. 1), intimacy negatively and conflicts are positively and significantly related to anxiety disorders [13]. In addition, many factors affect children's anxiety and hostility, such as genetics, life situations and parents' behaviors. Finally, the parents' behaviors can aggregate these symptoms, such as rejecting them and adopting hostile attitudes rather than accepting and establishing relaxing relationships [14].

**Fig. 2:**
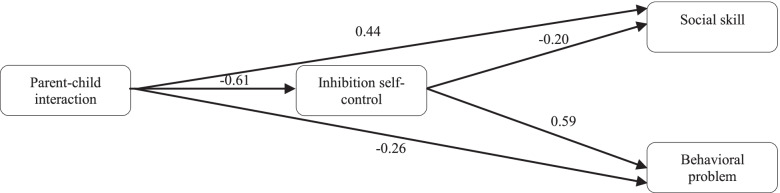
Relationships of interactions between parent–child relationships with behavioral problems in preschool children based on an inhibitory mediator [16]

As discussed earlier, the parents of these children resort to specific strategies to reduce their stress in the face of numerous problems. Adopting proper coping responses by parents significantly affects their child's relationship with behavioral problems [8, 15]. A fitted model introduced in Iran suggested the causal relationships of interactions between parent–child relationships with behavioral problems in preschool children based on an inhibitory mediator (2020) (Fig. 2) [16].

The factors associated with behavioral problems in these children include their age, disease duration, and the number of children in their family [8, 9]. However, the relationships between these factors, parent–child relationships and coping responses of parents with behavioral problems in children, all in one model, have rarely been addressed in the literature. Given the growing prevalence of this disorder in children and the importance of paying attention to this age group, the present study examined the parent–child relationship model and coping responses for behavioral problems in children with ADHD.

## Methods

### Study design and participants

This descriptive study was conducted in 2021 in Imam Ali and Imam Hossein psychiatric clinics affiliated with Alborz University of Medical Sciences in Karaj, the capital of Alborz Province, Iran's 4^th^ largest city and 22^nd^ most populous city in the Middle East.

According to a study by Keshavarz et al. [17] and the following formula, the sample size was estimated at 300 with a correlation coefficient of 0.16 between the score of the parent–child relationship and coping behaviors and type I and type II errors of 0.05 and 0.2, respectively.

$$c\,{=\frac{\mathrm{ln}\left(1+r\right)}{\mathrm{ln}\left(1-r\right)}n=\left(\frac{{Z}_{\alpha }+{Z}_{\beta }}{C}\right)}^{2}+3$$

Convenience sampling was performed until the sample was completed.

#### Inclusion criteria

##### Children

Children of both genders, aged 6–12 years, with a profile in the study clinics, diagnosed with ADHD by a psychiatrist as the study consultant and without other psychiatric disorders as confirmed by their parents were included.

According to the selection of the participants in the study, among the children who were referred to the clinic, the selection process was as follows:

First, all clients were evaluated using the Conners' Parent Rating Scale (CPRS) [17]. Then, all of them were assessed by an experienced psychiatrist using the Structured Clinical Interview for DSM Disorders (SCID) questionnaire regarding ADHD. After completing the diagnostic interview, if the final diagnosis was confirmed, the patients were treated with stimulants or other medication approved for treating ADHD patients. All patients under medical treatment were included in the study if the symptoms were partially or wholly controlled, without separating the participants according to the severity of the symptoms.

##### Parents

Literate Iranian parents with no psychological disorders based on the GHQ and no addiction to psychedelics and drugs were also included.

#### Exclusion criteria

Withdrawal from the study at the time of completing the questionnaires in a way that accessing the subject was impossible and leaving the questionnaires incomplete.

### Data collection and definition of terms

The data were collected using a demographic checklist, the child behavior checklist (CBCL), the parent–child relationship scale (PCRS), the Billings and Moos Coping Checklist, the socioeconomic status questionnaire and the general health questionnaire-28 (GHQ-28).

#### Demographic checklist

The demographic details of the children and their parents were obtained using a researcher-made questionnaire as the demographic- checklist included gender and age of the parents and child, marital status of the parents, child's weight, education and employment status of the parents, duration of current marriage of the parents, number of children, birth order of the affected child, ethnicity and alcohol consumption over the previous six months and smoking status in the parents.

#### Child behavior checklist (CBCL)

The present study employed the CBCL (Achenbach and Edelbrock)1991 to measure emotional-behavioural problems in children. This checklist measures emotional-behavioural problems [18], strengths and socio-educational competency in 6–18-year-olds from the perspective of their parents. This tool can be used in interviews and as a self-report instrument. The CBCL should be completed by the parents or guardians of the child. The items were scored on a three-point Likert scale ranging from 0 to two, with 0 denoting the absence of a behavior in the child, one denoting behaviors that occasionally emerge and two suggesting those that always or frequently occur. Eight problematic dimensions measured in children and adolescents using this questionnaire included anxiety/depression, isolation/depression, somatic complaints, social problems, thinking problems, attention problems, rule-breaking behaviors and aggressive behaviors. Normalizing this checklist in the Iranian population respectively obtained the retest validity coefficient and the internal consistency coefficient as 0.58 and 0.79 for the full competency scale, 0.58 and 0.83 for emotional-behavioural problems, 0.48 and 0.86 for internalizing problems, 0.97 and 0.88 for externalizing problems, 0.39 and 0.80 for attention deficits, 0.38 and 0.78 for thinking problems and 0.48 and 0.69 for social problems [19].

#### Parent–child relationship scale (PCRS)

Pianta introduced this 33-item scale in 1992 to measure the perception of parents of their relationship with their children in the subscales of conflicts (17 items), dependence (6 items), intimacy (10 items) and overall positive relationship. Conflicts involve negative aspects of the relationship such as conflict with each other, anger against each other, disobedience, rejection of restraint and unpredictability. Intimacy refers to the degree to which parents perceive their relationship with their child as warm, emotional and comfortable. Dependence assesses the degree of abnormal dependence of children on their mothers. The overall positive relationship also emphasizes intimate parent–child relationships. The PCRS is scored on a seven-point Likert scale with five denoting definitions applied as a self-report instrument. [20]. The reliability and validity of this scale were also confirmed in Iran. Moreover, the reliability of this tool was confirmed by calculating a Cronbach's alpha of 0.84 for conflicts, 0.69 for dependence and 0.80 for overall positive relationship [21].

#### Billings and moos coping checklist) coping responses)

Billings and Moos (1984) designed this 32-item checklist to investigate how individuals respond to stressful events and measure coping responses, including problem-focused coping (3 items), emotion-focused coping (11 items), coping based on cognitive evaluation (5 items), coping based on physical inhibition or somatization of problems (6 items) and coping based on achieving social support (4 items). The items were scored on a four-point Likert scale defined as 0: never, 1: sometimes, 2: often and 3: always [22]. The validity and reliability of this questionnaire were confirmed in Iran by calculating a Cronbach's alpha of 0.79 [23].

#### Socioeconomic status questionnaire

The socioeconomic status questionnaire(Ghodratnama), including five main and six demographic items, was used to evaluate four dimensions of socioeconomic status, i.e. parental education, income, economic class, and housing status, which are scored based on a Likert scale from 1 to 5 as 1: very low to 5: very high. Eslami et al. confirmed the face and content validity of this questionnaire in Iran (2014), and its reliability was confirmed by calculating a Cronbach's alpha of 0.83 [24].

#### General health questionnaire (GHQ)

Goldberg and Hiller (1979) designed this 28-item questionnaire with four 7-item subscales, i.e. somatic symptoms (items 1–7), anxiety and insomnia (items 8–14), social dysfunction (items 15–21) and severe depression (items 22–28). The items were scored on a scale of 0–3 with a cutoff point of 23. In other words, the higher 23 the score obtained from this questionnaire, the worse the mental health [25]. Najarkolaei et al. confirmed the validity and reliability of this instrument in Iran in 2014 by calculating a Cronbach's alpha of 0.85 [26].

### Procedure

The present study began after receiving the approval of the Ethics Committee of Alborz University of Medical Sciences (IR.ABZUMS.REC.1399.238). After presenting to Imam Ali and Imam Hossein psychiatric clinics affiliated with the university, the researcher identified eligible candidates, briefed them on the study objectives and asked their parents to sign written consent forms. To evaluate the mental health of the parents accompanying their children, they were asked to complete the GHQ. The parents receiving a score of below 23 from the GHQ, suggesting an absence of psychological problems, were included in the study. The questionnaires were completed online, given the limitations on the presence of the participants in the clinics caused by the COVID-19 pandemic and social distancing rules at the time of the study. The links to the questionnaires were sent to the parents, who were asked to complete them within two weeks. In case of inability to complete the questionnaires online, the PCRS, the coping responses questionnaire, the socioeconomic status questionnaire, the GHQ-28 and the demographic checklist were distributed in calm conditions among the parents, and they were assured of the confidentiality of their information. The researcher constantly monitored the procedure and responded in person to the potential queries of the participants completing the questionnaires at the clinic and through the phone to those communicating online. In case of inability to complete the questionnaires in one session, the participant was asked to participate in the following sessions at their convenience.

### Statistical analysis

**Fig. 3:**
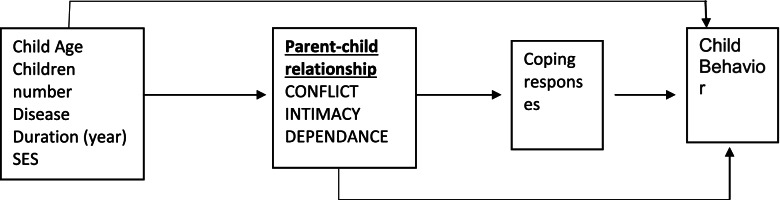
Conceptual relationship model among parent–child relationship, coping responses and behavioral problems in children with ADHD

Developing a conceptual model based on a review of the literature and previously-proposed models and theories constitutes the first step in testing the relationship model [27]. The parent–child relationship and coping responses of parents were therefore examined based on the conceptual model of relationship (Fig. 3). The distribution normality of the quantitative variables was investigated using the Kolmogorov–Smirnov test. As ordinary generalized regression, the path analysis was performed to explain direct and indirect relationships and effects of individual variables on dependent variables. The obtained results were used to interpret the relationships and correlations logically. The data were analyzed in SPSS-25 and LISREL 8.8. The Pearson correlation coefficient was used for investigating the correlations, and the path analysis was expressed as Standardized Beta with a significance level of T-value > 1.96.

## Results

The present study recruited 300 children aged 6–12 with ADHD and profiles in the selected clinics. The mean age of the children was 8.1 ± 2.4 years, and the duration of their disease was 2.9 ± 1.7 years. Mean scores obtained were 59.6 ± 29.8 for CBCL, 42.1 ± 9.7 for the coping responses, and 96.6 ± 13.6 for the positive score of the parent–child relationship (Table 1).

**Table 1:** Frequency distribution of individual-social characteristics of participants in 2021

Variables(quantitative)		Mean ± SD	minimum		maximum
Age child(year)		8.1 ± 2.4	3		13
Age mother		36.4 ± 7.2	20		56
Age father		40.3 ± 6.9	28		64
disease duration (year)		2.9 ± 1.7	1		10
Child-parents relation		96.6 ± 13.6	71		135
Child behavior problem		59.6 ± 29.8	4		136
Coping Strategies Scale		42.1 ± 9.7	17		67
Variables(qualitative)		F (%)	Variables		F (%)
Child birth rating	1	194(64.7)	Number of children	1	115(38.3)
	2	86(28.7)		2	147(49)
	≥ 3	20(6.7)		≥ 3	38(12.7)

The Pearson correlation test suggested the significant relationships between behavioral problems in children and variables of the number of children, conflicts, dependence, intimacy, positive parent–child relationship and coping responses. The highest positive correlation (r = 0.45) was related to positive parent–child relationships and the highest negative (r = -0.58) to behavioral problems.

**Fig. 4:**
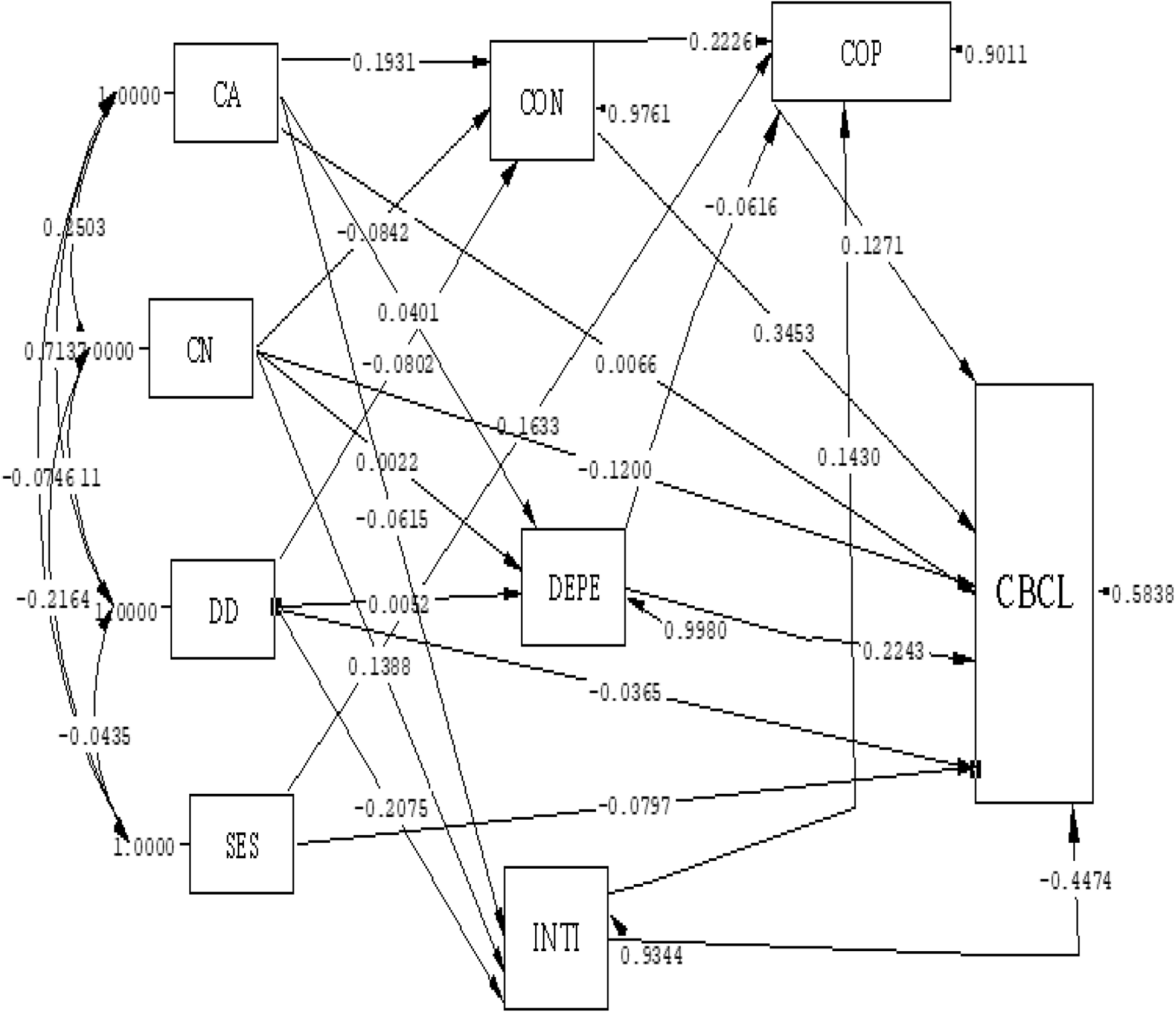
Path analysis in the relationship model among parent–child relationship domains, coping responses, and children's behavioral problems according Standard B. CON = conflict, DEPE = dependence, INTI = intimacy, COP = parents' coping responses, CBCL = Child behavior checklist, SES = Socio economic Statues, DD = Disease Duration, CN = Children number, CA = child Age

Based on the findings of the path analysis test on the relationship model among parent–child relationship domains, coping responses, and children's behavioral problems, the dependency domain from the parent–child relationship (B = 0.22) had the most positive causal effect on behavioral problems in children in the direct path such that an increase in the score of dependency increased children's behavioral problems. In the indirect path, disease duration (B = 0.085) exerted the highest positive causal effect on behavioral problems in the children. The longer the disease duration, the more frequent the behavioral problems. Among the effective variables in both direct and indirect paths, conflicts (B = 0.366) exerted the highest positive causal effect and intimacy (B = -0.42) the highest negative effect. Increases in conflicts and intimacy scores respectively increased and decreased behavioral problems in the children. The extent to which the parents used coping responses exerted positive causal effects on behavioral problems on the direct path (B = 0.12). In other words, increasing parental use of coping responses was associated with increased behavioral problems in the children (Table 2, Fig. 4).

**Table 2:** Direct and indirect effect of relationship model among parent–child relationship domains, coping responses and children's behavioral problems in children with ADHD

	Standard B			Un standard B		
Variables	Direct Effect	Indirect effect	total	Direct Effect	Indirect effect	Total
Child age	0.006	0.07*	0.07*	0.086	0.97*	0.97*
Children number	-0.12*	-0.055*	-0.17*	-4.79*	-2.36*	-7.15*
disease duration	-0.036	0.085*	0.085*	-0.66	1.61*	1.61*
Socioeconomic status	-0.07	0.019*	0.019*	-1.187	0.304*	0.304*
coping responses Scale	0.12*	-	0.12*	0.40*	-	0.40*
conflicts	0.34*	0.026*	0.366*	0.99*	2.32*	3.31*
dependence	0.22*	-0.007	0.22*	2.33*	-0.08	2.33*
Intimacy	-0. 44*	0.016*	-0. 42*	-4.33*	0.172*	-4.158*

**Fig. 5:**
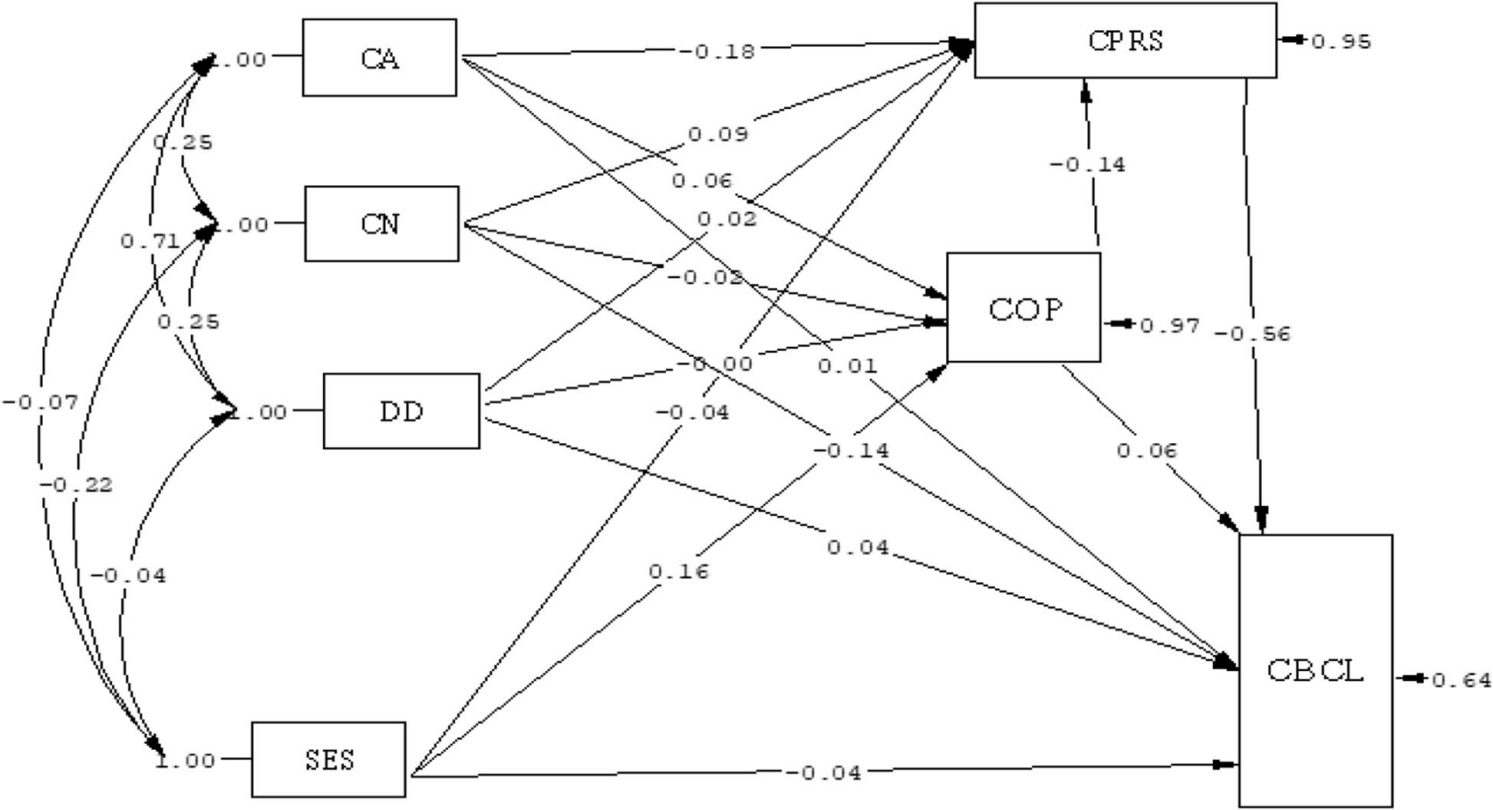
Path analysis in the relationship model among positive parent–child relationship, coping responses and children's behavioral problems according Standard B. CPRS = positive Child-Parent Relationship Scale, COP = parents' coping responses, CBCL = Child behavior checklist, SES = Socio economic Statues, DD = Disease Duration, CN = Children number, CA = child Age

According to the findings of the path analysis in the relationship model among positive parent–child relationship, coping responses and children's behavioral problems, the positive score for the parent–child relationship had the most negative causal relationship (B = -0.56) in the direct path. The child's age had the most positive causal relationship (B = 0.1) with behavioral problems in the indirect path. As such, the higher the positive parent–child relationship score, the fewer the children's behavioral problems, and the older the child, the more behavioral problems the children have. The use of coping responses was indirectly and positively associated with behavioral problems (B = 0.07) (Table 3, Fig. 5).

**Table 3:** Direct and indirect effect of relationship model among positive parent–child relationship, coping responses and children's behavioral problems in children with ADHD

	Standard B			Un standard B		
Variables	Direct Effect	Indirect effect	total	Direct Effect	Indirect effect	Total
child age	0.01	0.1*	0.1*	0.12	1.35*	1.35*
Children number	-0.14*	-0.051	-0.14*	-5.88*	-0.051	-5.88*
disease duration	0.04	-0.011	0.029	0.69	-0.003	0.687
Socioeconomic status	-0.04	0.012*	0.012*	-0.62	0.18*	0.18*
coping responses Scale	0.06	0.07*	0.07*	0.19	0.25*	0.25*
positive parent–child relationship	-0.56*	-	-0.56*	-1.32*	-	-1.32*

The fitted model indices showed the desirability, high model fit and rationality of the adjusted relationships of the variables based on the conceptual model. The fitted model was insignificantly different from the conceptual model (Table 4).

**Table 4:** The fitted model index

	X^2^	df	X^2^/df	NFI	NNFI	CFI	GFI	AGFI	RMSEA
Fitted model index with parent–child relationship domains	5.55	5	1.11	0.91	0.92	0.98	0.99	0.96	0.01
Fitted model index with among positive parent–child relationship	3.93	4	0.98	0.99	1	1	1	0.97	0.000
Standard	X^2^/df < 5			> 0.90	> 0.90	> 0.90	> 0.90	> 0.90	< 0.05

## Discussion

Psychologists have always addressed ADHD as a prevalent disorder with negative consequences for children. Therefore, the present research examined the parent–child relationship model and strategies to cope with behavioral problems in children with ADHD.

According to the results of the path analysis of the relationship model among parent–child relationship domains, coping responses, and children's behavioral problems, among variables that had the most casual relationship with children's behavioral problems, the parent–child dependency domain from the parent–child relationship had the most positive relationship. In other words, the children's behavioural problems increased as dependence on the parent–child relationship increased. Varasteh et al. (2016) reported a positive parenting program's positive and significant effects on conflicts, intimacy, dependence and overall positive effect. They reported less frequent behavioral problems in children with decreased dependence and conflicts in the parent–child relationship [28]. According to Adili et al. (2019), using intervention methods such as play therapy decreases dependence on the parent–child relationship, significantly reduces conflicts and improves children's health dimensions [29]. The excessive emotional care and attention received by these children appear to intensify dependence on their parents, increase their expectations and create different problems in their future, which is consistent with the present findings.

The disease duration was positively, significantly and indirectly related to behavioral problems in the children on a single path. Increases in the disease duration increased behavioral problems in the children. As a major daily issue in patients with ADHD, establishing social relationships is exacerbated with age [30]. Attention and concentration problems persist despite the gradual improving effect of age on the symptoms of ADHD, as reflected in numerous studies [29]. In line with the present research, a study by Sohrabi et al. (2014) on oppositional defiant disorder in preschool children showed no gradual changes in the symptoms of disobedience in the controls. This disorder persisted over time [31].

Among the influential variables on both paths, conflicts exerted the highest positive causal effect on behavioral problems in children. An increase in conflicts in the parent–child relationship increases the behavioral problems. Shafiei et al. (2018) found conflicts in parent–child relationships to cause externalizing disorders in children. They also found treatments focusing on sensory processing patterns in children with behavioral-emotional problems, training their parents in individual differences, resolving conflicts, and increasing positive interactions to decrease problems in these patients [32]. Shapurabadi et al. (2012) found designing positive group parenting programs for the mothers of children with ADHD significantly reduces conflicts and dependence, increases intimacy and improves mother–child relationships [33]. Memarbashi et al. (2020) found inhibitory control to mediate the relationships of parent–child interactions with social skills and behavioral problems. In other words, parent–child interactions were found to relate to social skills and behavioral problems both directly and indirectly through inhibitory control. Disabilities and lack of concentration in these children cause their failure to complete their normal tasks, increase pressure on their parents in terms of supervision and therefore create conflicts in parent–child relationships [34].

As a dimension of parent–child relationships, intimacy exerted the highest negative effect on behavioral problems in the children in both direct and indirect paths. In fact, the higher the intimacy score, the lower the behavioral problems in the children. A study by Abbasi et al. (2017) on the mother–child relationship model and the effects of positive and negative emotions on anxiety disorders showed that, among the dimensions of parent–child relationships, intimacy was negatively and significantly, and conflicts positively and significantly related to anxiety disorders. Conflicting behaviors in a mother toward their children decrease their sense of responsibility and develop their insecurity, anxiety, isolation, carelessness and submissiveness. In contrast, increasing parent–child intimacy reduces children's anxiety disorders and behavioral problems. In other words, parent–child relationships based on intimacy and empathy create joy and positive emotions in children. Conversely, parent–child relationships based on conflicts create doubts about parental support, undesirable feelings and negative emotions in children [13].

The path analysis suggested positive relationships between coping responses used by parents and behavioral problems in their children. Research suggests properly using coping responses by parents significantly affects the quality of their relationship with and behavioral problems in their child [8, 15]. Hojjati et al. (2018) found behavioral problems to increase stress in children through increasing fatigue and ineffective coping responses in mothers. Fatigue and lack of energy in mothers associated with their children's behavioral problems cause their adoption of ineffective, emotion-driven and avoidance coping responses, exacerbating their child's behavioural problems [35].

Based on the test results in the relationship model among positive parent–child relationships, coping responses and children's behavioral problems, a positive parent–child relationship had the most negative causal relationship with the child's behavioral problems. In other words, increases in the parent–child relationship's positive score decreased the children's behavioural problems. This finding can be explained by the significant effects of positive parent–child relationships on improving the mother–child relationship, reducing conflicts and dependence and increasing intimacy [36]. Research suggests parenting skills and familiarity of parents with training methods and efficient communication with children exert the most significant effect on children's emergence or persistence of behavioral problems. Positive parent–child interactions can modify this bad relationship and help improve behavioral problems in children [37, 38]. According to Pajooh et al. (2018), mothers' familiarity with the nature of behavioral problems in their children, their increased awareness of communication skills and their change in inefficient parent–child patterns to reduce behavioral problems in their children, improve their understanding of their children's behavior and help them more effectively communicate with their children [39].

The child's age indirectly exerted the highest positive causal effect on behavioral problems. Research suggests associations between ADHD and socio-behavioral problems in children. Untreated ADHD can also persist until adolescence and adulthood [40]. Despite the improving effect of age on behavioral problems in children, recovery before the age of 12 years is unlikely, and symptoms persist until adulthood in 15–20% of cases [41]. The positive and significant causal relationship observed between the children's age and their behavioral problems in the present study can be explained by their age being below 12 years. Khoddam et al. (2009) reported varying symptoms with age and observed the highest incidence in children aged at most 11 years [42]. The symptoms begin to emerge in 67% of children with ADHD at preschool age [43]. Research suggests the maximum prevalence of this disorder in children aged 3–5 years. Failing to timely diagnose and treat this disorder, especially in acute cases with social problems, can prolong the condition until the following stages of life [44, 45].

### Limitation

The present study's limitations included using questionnaires for recording data and recruiting school-age children with this disorder, as in the case of numerous similar studies that failed to address preschool children. The other limitations of the COVID-19 pandemic and social distancing rules included limited face-to-face contact with the parents. Another limitation of the present study was the lack of separation of the participants in terms of the severity of the symptoms and the extent to which the symptoms were under control at the time of the study. In this case, in future studies, data analysis based on the difference in the severity of symptoms and the degree of symptom control can be considered. This study didn't evaluate the comorbidities with ADHD. Still, because we use Path analysis based on multiple regression techniques, all confounders are adjusted and don't affect the results, which can be our limitation.

## Conclusion

The present findings suggested significant causal relationships between the parent–child relationship and the extent to which the parents used coping responses to deal with behavioral problems in their children. Therefore, it is recommended that ADHD be diagnosed before the school age and entrance to social settings, that mothers be familiarized with the nature of behavioral problems in their children and that training programs be developed to improve communication skills, coping responses and problem-solving skills in mothers.

## Data Availability

The data that support the findings of this study are available from the corresponding author upon reasonable request.

## Abbreviations

CON: Conflict
DEPE: Dependence
INTI: Intimacy
COP: Parents' coping responses
CBCL: Child behavior checklist
SES: Socio economic Statues
DD: Disease duration
CN: Children number
CA: Child age
CPRS: Positive child-parent relationship scale
